# Impact of colistin plasma levels on the clinical outcome of patients with infections caused by extremely drug-resistant *Pseudomonas aeruginosa*

**DOI:** 10.1186/s12879-016-2117-7

**Published:** 2017-01-05

**Authors:** Luisa Sorlí, Sonia Luque, Concepción Segura, Nuria Campillo, Milagro Montero, Erika Esteve, Sabina Herrera, Natividad Benito, Francisco Alvarez-Lerma, Santiago Grau, Juan Pablo Horcajada

**Affiliations:** 1 Infectious Disease Service, Hospital del Mar, Barcelona, Spain; 2 Pharmacy Service, Hospital del Mar, Barcelona, Spain; 3 Institut Hospital del Mar d’Investigacions Mèdiques (IMIM), Barcelona, Spain; 4 Laboratori de Referència de Catalunya, Prat de Llobregat, Spain; 5 InfectiousDiseaseUnit, Department of Internal Medicine, Hospital de la Santa Creu i Sant Pau, Barcelona, Spain; 6 Institut d’Investigació Biomèdica Sant Pau, Barcelona, Spain; 7 Spanish Network for Research in Infectious Diseases (REIPI RD12/0015), Instituto de Salud Carlos III, Madrid, Spain; 8 Intensive Care Unit, Hospital del Mar, Barcelona, Spain; 9 CIBERES, Madrid, Spain; 10 Universitat Autònoma de Barcelona, Barcelona, Spain; 11 CEXS-Universitat Pompeu Fabra, Barcelona, Spain

**Keywords:** Colistin, Mortality, Plasma concentration, *Pseudomonas aeruginosa*, Extremely drug-resistant, Nephrotoxicity

## Abstract

**Background:**

Colistin has a narrow therapeutic window with nephrotoxicity being the major dose-limiting adverse effect. Currently, the optimal doses and therapeutic plasma levels are unknown.

**Methods:**

Prospective observational cohort study, including patients infected by colistin-susceptible *P. aeruginosa* treated with intravenous colistimethate sodium (CMS). Clinical data and colistin plasma levels at steady-state (C_ss_) were recorded. The primary and secondary end points were clinical cure and 30-day all-cause mortality.

**Results:**

Ninety-one patients were included. Clinical cure was observed in 72 (79%) patients. The mean (SD) C_ss_ was 1.49 (1.4) mg/L and 2.42 (1.5) mg/L (*p* = 0.01) in patients who achieved clinical cure and those who not, respectively. Independent risk factors for clinical failure were male sex (OR 5.88; 95% CI 1.09–31.63), APACHE II score (OR 1.15; 95% CI 1.03–1.27) and nephrotoxicity at the EOT (OR 9.13; 95% CI 95% 2.06–40.5). The 30-day mortality rate was 30.8%. Risk factors for 30-day mortality included the APACHE II score (OR 1.98; 95% CI 1–1.20), the McCabe score (OR 2.49; 95% CI 1.14–5.43) and the presence of nephrotoxicity at the end of treatment (EOT) (OR 3.8; 95% CI 1.26–11.47).

**Conclusion:**

In this series of patients with infections caused by XDR *P. aeruginosa* infections, C_ss_ is not observed to be related to clinical outcome.

## Background

The increasing incidence of infections due to multidrug-resistant (MDR) gram-negative bacteria (GNB) is a concern worldwide due to high morbidity and mortality [1] and the lack of effective antimicrobials in the drug-development pipeline [2]. Of particular concern are MDR *Pseudomonas aeruginosa, Acinetobacter baumannii* and *Klebsiella pneumoniae* [3]. For infections with these organisms, the only therapeutic option may be colistin, which was discovered in the 1940s but never subjected to contemporary drug-development procedures. Colistin exhibits concentration-dependent bacterial killing, and its therapeutic efficacy depends on the ratio of the peak level to the minimal inhibitory concentration (MIC) or the area under the curve (AUC) to the MIC [4–6]. Colistin has a narrow therapeutic window, with nephrotoxicity being the most important dose-limiting adverse effect [4, 7]. Some non-comparative studies have reported that colistin has an acceptable efficacy and safety profile in the treatment of infections caused by MDR-GNB [8–10]. However, lower success rates are shown when compared with alternatives [11]. Although some clinical factors have been proposed as possible causes of this lower success rate, one important issue in this scenario is the shortage of available pharmacokinetic and pharmacodynamic data to guide the proper use of colistin in daily clinical practice [12].

Some recent studies have demonstrated that the current colistimethate sodium (CMS) dosage regimens are associated with suboptimal colistin concentrations and suboptimal pharmacokinetic targets for many strains of GN-GNB, and limited information is available regarding the optimal doses and the levels of colistin in plasma that provides a therapeutic effect. In addition, to date, no clinical studies have assessed the possible relationship between plasma colistin concentrations and clinical outcome. The aim of this study was to assess the possible relationship between colistin plasma concentration (C_ss_) and clinical cure and 30-day all-cause mortality in patients with infections due to colistin-susceptible extremely drug-resistant (XDR) *P. aeruginosa* who had been treated with intravenous CMS. The presence of nephrotoxicity at the end of treatment (EOT) was also analyzed.

## Methods

### Study population and data collection

A prospective observational cohort study was conducted between January 2009 and January 2013 at a 450-bed teaching hospital in Barcelona, Spain. All patients who had microbiologically documented infections due to colistin-susceptible XDR-*P.aeruginosa* and were administered CMS for at least 72 h, were included in the study. Only microbiologically documented and clinically defined infections caused by colistin-susceptible XDR *P. aeruginosa* treated with colistin were considered. Acute bronchitis and tracheitis were excluded from the analysis due to the difficulty in differentiating true infection from colonization in patients with chronic obstructive pulmonary disease (COPD).

Patients were identified through the hospital’s pharmacy registry of antibiotic use. Patients were excluded if they were < 18 years old, pregnant, breastfeeding, on renal replacement therapy prior CMS treatment or if they had received CMS treatment for less than 72 h. Patients who after a cured infection episode received another CMS curse due to another infection by XDR-*P. aeruginosa,* were considered as 2 different cases.

Patients were followed until hospital discharge or death.

The ethical committee of the hospital (Comité Ètic de Investigació Clínica del Parc de Salut Mar) approved the study. Informed consent was obtained form all participating patients or their legal representatives.

A standardized form was used to record patient characteristics, including age, sex, body weight, body mass index, data on the indication for CMS, the CMS administration schedule, the daily CMS dose in millions of international units (IU), the cumulative CMS dose (in millions of IU), the duration of treatment and the colistin plasma concentration at steady state (C_ss_). Additional clinical data collected for the study included the following: baseline glomerular filtration rate (GFR), calculated using the abbreviated Modification of Diet in Renal Disease equation (MDRD) [13]; the presence of chronic kidney disease (CKD) at baseline, which was diagnosed when the GFR was < 60 mL/min/1.73 m^2^ for ≥ 3 months; the presence of underlying comorbidities, evaluated by the Charlson comorbidity index [14]; the severity of disease at the time of the first CMS dose, stratified according to the Acute Physiology and Chronic Health Evaluation (APACHE II) [15]; concomitant nephrotoxic drugs (aminoglycosides, vancomycin, nonsteroidal anti-inflammatory drugs, intravenous radiocontrast agent, loop diuretic, angiotensin-converting-enzyme inhibitors, angiotension II receptor antagonist and ciclosporine); the causative organism and in vitro susceptibility data; the use of combined antibiotic treatment; and the clinical response to therapy. Data were prospectively collected for 30 days from the onset of CMS treatment, unless otherwise specified.

The primary end point was clinical cure, and the secondary 30-day all-cause mortality.

### Definitions and microbiological testing

Infections were defined according to the Centers for Disease Control and Prevention (CDC) [16]. The patient’s clinical status at the beginning of CMS treatment was defined as infection, severe sepsis or septic shock, According to standard definitions [17], severe sepsis was considered when an acute organ dysfunction secondary to documented or suspected infection and septic shock was defined as having a severe sepsis plus hypotension not reversed with fluid resuscitation.

Clinical cure and failure were defined as resolution and persistence/worsening, respectively, of symptoms and signs of infection.

Thirty-day all-cause mortality was considered as death during the hospitalization.

XDR-*Pseudomonas aeruginosa* was defined according to the CDC/ECDC criteria [18] as an isolate non-susceptible to at least 1 agent in all but 2 or fewer categories: aminoglycosides, antipseudomonal carbapenems, antipseudomonal cephalosporins, antipseudomonal fluoroquinolones, antipseudomonal penicillins + *β-lactamase* inhibitor, monobactams, phosphonic acids and polymyxin.

Combined antibiotic treatment consisted of CMS plus ceftazidime or carbapenem in high-dose extended infusion, or CMS plus amikacin.

Routine identification and susceptibility testing of causative microorganisms were first performed by microdilution using the gram-negative (GN) breakpoint panel for non-fermenting GNB of the MicroScan® WalkAway system (Siemens Diagnostic Inc., CA). The colistin Minimal Inhibitory Concentration (MIC) was determined by microdilution using cation-adjusted MHB; the isolates were considered susceptible if the MIC value was < 2 mg/L according to the Clinical and Laboratory Standards Institute (CLSI). Performance standards for antimicrobial susceptibility testing; informational supplement. CLSI document M100-SCLSI, Wayne, PA (2013).

### CMS administration and colistin plasma level measurements

CMS for injection was used and dosed in millions of IU throughout the study. The drug was administered intravenously in 100 mL of normal saline over 30 min in the commercially available colistimethate formulation for injection (GES GenéricosEspañoles®), with each vial containing 80 mg CMS (equivalent to 1 million IU of CMS and about 30 mg of colistin base activity). As the policy of our hospital, CMS is reserved for the treatment of infections due to bacteria that are resistant to carbapenems and other antipseudomonal antibiotics except colistin and, eventually, aminoglycosides. Dose selection was at the discretion of the responsible clinicians. CMS doses ranged from 1 to 3 million IU every 8 h (3–9 millions IU daily). The following dose adjustments were made according to the package insert’s recommendations in patients with impaired renal function: GFR ≥ 76 mL/min/1.73 m^2^, 4–6 million IU daily in three doses; GFR 40 to 75 mL/min/1.73 m^2^, 2–3 million IU daily in 2 doses; GFR 25–40 mL/min/1.73 m^2^, 1.5–2 million IU daily divided into 1 or 2 doses; and GFR < 25 mL/min/1.73 m^2^, 0.6–1 million IU daily every 36 h. No loading-dose was administered to any included patient.

Measurement of the plasma colistin concentration was performed at the fourth day of treatment, when was assumed that the colistin concentrations had reached the steady state. The colistin plasma trough concentrations (C_min_) were measured just before the administration of CMS. The maximum plasma concentrations (C_max_) were obtained 30 min after the end of the CMS infusion (1 h after the start of infusion). A specialized nurse performed extraction of blood. Samples were immediately placed in a portable refrigerator (4 °C) to be processed in a refrigerated centrifuge (4 °C) at the laboratory. Colistin concentrations in the plasma were determined using a validated high-performance liquid chromatography (HPLC) method as reportedby Li et al. [19], with minor modifications as described previously by our group [20].

Because the C_max_ (1.66 ± 1.40 mg/L) and C_min_ (1.71 ± 1.42 mg/L) were highly correlated (*R* = 0.98; *P* < 0.001), only one of these variables was used throughout the study as a marker of colistin plasma exposure. Following Couet et al. [21], C_min _was chosen because it is more convenient from a practical view point and because due to the colistin pharmacokinetic behavior, there can be CMS hydrolysis to colistinafter sample extraction resulting in an overestimation of the real in vivo colistin plasma concentration. This phenomenon can be minimized when CMS plasma concentrations are lower what occurs at the end of the dosing interval (when the C_min_ is measured). Throughout the present paper, we have used the term “average colistin concentration at steady state (C_ss_)” to refer to the C_min_.

### Efficacy and nephrotoxicity assessment

Thirty-day mortality was defined as death occurring within 30 days of beginning CMS treatment.

Clinical cure was defined as having resolved clinical signs and symptoms and/or no need for additional antibiotic therapy. Clinical failure was defined as an inadequate response to antibiotic therapy, with worsening, new/recurrent signs and symptoms or death.

The serum creatinine level and estimated glomerular filtration rate (GFR) were recorded at baseline, at the moment of colistin plasma extraction (day 4), on day 7 and at the EOT. We chose these time points because in our center, blood tests are performed routinely in all inpatients at least once a week and at the end of treatment. The RIFLE criteria (Table 1) estimated with exclusion of the urinary output criterion, were used for the detection and stratification of acute kidney injury (AKI) [22]. AKI during CMS treatment was defined as a 1.5-fold or more increase in serum creatinine and/or a decrease in the GFR of 25% or more. These criteria needed to be fulfilled for at least 2 consecutive measurements 24 h apart during CMS treatment.

**Table 1:** Definition of the RIFLE criteria to assess renal injury

Category criteria	Definition
Risk (R)	Increased creatinine level × 1.5 or ^1^﻿GFR decrease > 25%
Injury (I)	Increased creatinine level × 2 or ^1^﻿GFR decrease > 50%
Failure (F)	Increased creatinine level × 3, ^1^﻿GFR decrease > 75%, or creatinine level > 4 mg/dL
Loss (L)	Persistent acute renal failure or loss of function for > 4 weeks
End-Stage Kidney Disease (ESKD) (E)	ESKD for > 3 months

^1^ *GFR* glomerular filtration rate

### Statistical analysis

Dichotomous data were compared using a χ^2^ or Fisher’s exact test. Normally distributed continuous data are expressed as the means with standard deviations (SD) and were compared using the *t*-test. Otherwise, values are presented as the means with interquartile range (IQR) and were compared using the Mann–Whitney *U*-test.

Logistic regression was used to explore risk factors associated with clinical cure and 30-day all- cause mortality.

Univariate analyses were performed separately for each of the risk factor variables to ascertain the odds ratios (ORs) and 95% confidence intervals (CI). For continuous variables found significant in the analysis, the threshold breakpoint was determined (C_ss_). All clinically important covariates and those with a *P* value < 0.2 in the univariate analyses were included in the multivariate analysis.

C_ss_ was also analyzed and included in the model as a dichotomous variable by defining one set point. According to Garonzik et al. [6], this set point was 1.25 mg/L, corresponding to a target AUC_0–24_ of 60 mg.h/L for a *P. aeruginosa* strain with an MIC value of 0.5 mg/L, which is the predominant strain at our center.

A backward selection process was utilized in which the results of the Wald test for individual parameters were examined. The least significant effect that did not meet the level for staying in the model was removed and it remained excluded.

Multivariate logistic regression models were assessed using the Hosmer–Lemeshow goodness-of-fit test. The discriminatory power of the predictive model was established by means of receiver operating characteristic (ROC) curves. For all analyses, a two-sided *P* value <0.05 was considered to be statistically significant. The Statistical Package for the Social Sciences (SPSS, version 15.0) was used for the statistical analysis.

## Results

During the study period, 124 patients with colistin-susceptible XDR *P. aeruginosa* infections treated with intravenous CMS were evaluated. Of these, 33 (26.6%) were diagnosed with acute bronchitis or tracheitis and were excluded. Thus, 91 patients were finally included in the analysis. Three patients developed 2 infectious episodes due to XDR-*P. aeruginosa* (patient 1: two episodes of UTI in 3 months. The second episode was considered a new infection; patient 2: two episodes of UTI in 5 months. The second episode was considered a new infection; patient 3: one episode of ventriculitis and one UTI), and each infection was included as a separate case. The clinical and demographic characteristics of the included patients are shown in Table 2.

**Table 2:** Clinical and demographic characteristics of included patients

	Included patients (*n* = 91)
Age, years*	67 (24–88)
Male sex, *n* (%)	66 (72.5)
APACHE II*	11 (2–28)
Co-morbidities, *n* (%):	
Malignancy	14 (15.4)
Cardiovascular	26 (28.6)
Pulmonary	33 (36.3)
Diabetes Mellitus	21 (23.1)
Urogenital^a^	14 (15.4)
Hepatic	8 (8.8)
Haematological^b^	12 (13.2)
Neurological^c^	18 (19.2)
Charlson score*	4 (0–10)
McCabe score**	1.48 ± 0.64
Patients with CKD^1^ at baseline	19 (20.9)
Type of infection, *n* (%):	
Pneumonia	24 (24.6)
Urinary tract infection	22 (24.2)
Skin and soft tissue infection	11 (12.1)
Organ space surgical site infection	10 (11)
Bacteremia	6 (6.6)
Other	18 (19.8)
Hospital-acquired infection, *n* (%)	86 (94.5)
Department of hospitalization:	
Medical	44 (48.2)
Surgical	32 (35.2)
ICU^2^	15 (16.2)
Admission diagnosis category:	
Infection	26 (28.6)
Other-medical	35 (38.5)
Other-surgical	30 (33)
CMS^3^ daily dose (millions of IU^4^)**	5.45 ± 2.21
CMS^3^ total dose (millions of IU^4^)**	108.36 ± 96.41
CMS^3^ duration of treatment, days**	20.18 ± 16.01
Inhaled CMS^3^, *n* (%)	14 (15.4)
Combined antimicrobial therapy, *n* (%)	46 (50.5)
C_ss_^5^ (mg/L)**	1.67 ± 1.42
C_ss_^5^ > 1.28 (mg/L), *n* (%)	46 (50.5)
C_ss_^5^/MIC^6**^	3.43 ± 2.91
AKI^7^ prior to CMS^3^ treatment, *n* (%)	12 (13.2)
Patients with AKI at day 7, *n* (%)	
R (Risk)	19 (20.9)
I (Injury)	9 (9.9)
F (Failure)	2 (2.2)
Patients with AKI^7^ at the EOT^8^, *n* (%)	
R (Risk)	12 (13.2)
I (Injury)	27 (27.7)
F (Failure)	10 (11)
Clinical response, *n* (%)	72 (79.1)
30-Day all-cause mortality, *n* (%)	28 (30)
Hospital length-of-stay (days)*	67 ± 53.97

^1^ *CKD* chronic kidney disease, ^2^ *ICU* intensive cure unit, ^3^ *CMS* colistin methanesulphonate, ^4^ *IU* international units, ^5^ *C*_*ss*_ colistin plasma concentration at steady-state, ^6^ *MIC* minimal inhibitory concentration, ^7^ *AKI* acute kidney injury, ^8^ *EOT* end of treatment

*median (range)

**mean ± SD

^a^Among urogenital co-morbidities were, renal disease, kidney stones and obstructive uropathy

^b^Among haematological co-morbidities were haemopoietic and lymphoreticular malignances

^c^Among neurological co-morbidities were Alzheimer’s disease, stroke, miastenia gravis, sclerosis and any kind of dementia

Clinical cure was observed in 72 (79.1%) patients. Table 3 shows the clinical characteristics of patients with clinical cure and clinical failure. The patients with clinical failure were mainly men with higher APACHE II indices. Additionally, they had achieved higher C_ss_ values and were more likely to have developed AKI than patients with clinical cure. In the multivariate analysis, factors related to clinical failure were male sex (OR 5.88; 95% CI 1.09–31.63, *P* = 0.039), APACHE II score (OR 1.15; 95% CI 1.03–1.27, *P* = 0.013) and the presence of AKI at the EOT (OR 9.13; 95% CI 95% 2.06–40.5, *P* = 0.004).

**Table 3:** Clinical and demographic characteristics of patients with and without clinical cure

	Clinical cure	Clinical failure	*P*-value
	(*n* = 72)	(*n* = 19)	
Male sex	49 (68)	17 (89.5)	0.06
Age, years*	66.5 (24–88)	67 (41–84)	0.59
APACHE II*	11 (2–28)	13.5 (6–24)	0.05
Co-morbidities, *n* (%):			
Malignancy	11 (15.3)	3 (15.8)	1
Cardiovascular	21 (29.2)	5 (26.3)	1
Pulmonary	24 (33.3)	9 (47,4)	0,26
Diabetes Mellitus	20 (27.8)	1 (5.3)	0.06
Urogenital^a^	11 (15.3)	3 (15.8)	1
Hepatic	8 (11.1)	8 (0)	0.2
Haematological^b^	9 (12.5)	3 (15.8)	0.7
Neurological^c^	16 (22.2)	2 (10.5)	0.34
Charlson*	4.5 (0–10)	4 (1–9)	0.73
McCabe**	1.4 ± 0.6	1.7 ± 0.7	0.11
Severe sepsis, *n* (%)	49 (68.1)	9 (47.4)	0.095
Shock	6 (8.3)	1 (5.3)	0.65
Patients with CKD at baseline	16 (22.2)	3 (15.8)	0.53
Department of hospitalization:			
Medical	38 (52.8)	6 (31.6)	0.16
Surgical	23 (32)	9 (47.4)	
ICU^2^	11 (15.3)	4 (21.1)	
Admission diagnosis category:			
Infection	19 (26.4)	7 (36.8)	
Other-medical	30 (41.7)	5 (26.3)	
Other-surgical	23 (3.9)	7 (36.8)	0.45
CMS daily dose (millions IU)**	5.3 ± 2.3	6.2 ± 2.1	0.094
CMS total dose (millions IU)**	105.91 ± 88.9	141.2 ± 129.5	0.45
CMS duration of treatment, days**	20.66 ± 16.1	22.6 ± 18.5	0.81
Combined treatment, *n* (%)	35 (48.6)	11 (57.9)	0.47
C_ss_ (mg/mL)**	1.49 ± 1.4	2.42 ± 1.49	0.01
C_ss_ > 1.25 (mg/mL), *n* (%)	32 (45.1)	14 (73.7)	0.027
C_ss_/MIC**	3.13 ± 2.9	4.61 ± 2.86	0.03
AKI at day 7, *n* (%)	18 (25)	12 (63.2)	0.002
AKI at the EOT, *n* (%)	33 (45.8)	16 (84.2)	0.003
Length of stay, days**	69.16 ± 59.5	70.8 ± 39.1	0.19

^1^ *CKD* chronic kidney disease, ^2^ *ICU* intensive cure unit, ^3^ *CMS* colistin methanesulphonate, ^4^ *IU* international units, ^5^ *C*_*ss*_ colistin plasma concentration at steady-state, ^6^ *MIC* minimal inhibitory concentration, ^7^ *AKI* acute kidney injury, ^8^ *EOT* end of treatment

*median (range)

**mean ± SD

^a^Among urogenital co-morbidities were, renal disease, kidney stones and obstructive uropathy

^b^Among haematological co-morbidities were haemopoietic and lymphoreticular malignances

^c^Among neurological co-morbidities were Alzheimer’s disease, stroke, miastenia gravis, sclerosis and any kind of dementia

Twenty eight patients died during hospitalization so the 30-day all-cause mortality rate was 30.8%. However, only 13 patients died due to *P.* aeruginosa infection leading to a infection-related mortality rate of 14.3%. These data reveals that 15 out of the 28 patients who died (53.6%) did so for reasons other than the infection itself. Causes of death in these patients were mainly related to their comorbid conditions (4 patients of COPD complications, 2 of cirrhosis, 2 of heart failure, 1 because malignancy, 2 of hematological malignancies, 1 because complications of *Clostridium difficile* infection) or post-surgical complications (3 patients). So only one death in this group could be related to colistin treatment and it was one patient who died because a *Clostridium difficile* infection. However, this patient has also been treated with other antibiotics in addition to colistin. The patients who died were mainly men with higher Charlson, APACHE II and McCabe scores, higher C_ss_ values and a higher likeliness of having developed AKI during treatment (Table 4). In the multivariate analysis, the following independent risk factors for 30-day mortality were identified: APACHE II score (OR 1.98; 95% CI 1–1.20, *P* = 0.046), McCabe score (OR 2.49; 95% CI 1.14–5.43, *P* = 0.021), and the presence of AKIat the EOT (OR 3.8; 95% CI 1.26–11.47, *P* = 0.018).

**Table 4:** Clinical and demographic characteristics of patients who died and those who survived

	Died (*n* = 28)	Survived (*n* = 63)	*P*-value
Male sex	24 (85.7)	42 (66.7)	0.06
Age, years*	66.85 (41–84)	65.5 (24–87)	0.12
APACHE II*	14 (5–27)	10.5 (2–28)	0.047
Co-morbidities, *n* (%):			
Malignancy	6 (21.4)	8 (12.7)	0.35
Cardiovascular	7 (25)	19 (30.2)	0.8
Pulmonary	15 (53.6)	18 (28.6)	0.03
Diabetes Mellitus	3 (10.7)	18 (28.6)	0.1
Urogenital^a^	6 (21.4)	8 (12.7)	0.35
Hepatic	4 (14.3)	4 (6.3)	0.24
Haematological^b^	4 (14.3)	8 (12.7)	1
Neurological^c^	2 (7.1)	16 (23.4)	0.05
Charlson*	5 (1–9)	4 (0–10)	0.039
McCabe**	1.8 ± 0.7	1.3 ± 0.6	0.008
Clinical status, *n* (%)			
Severe sepsis	15 (53.6)	43 (68.3)	0.17
Shock	2 (7.1)	5 (7.9)	0.89
Patients with CKD at baseline	7 (25)	12 (19)	0.52
Department of hospitalization:			
Medical	12 (42.9)	32 (50.8)	
Surgical	11 (39.3)	21 (33.3)	0.78
ICU^2^	5 (17.9)	10 (15.9)	
Admission diagnosis category:			
Infection	7 (25)	19 (30.2)	
Other-medical	12 (42.9)	23 (36.5)	0.82
Other-surgical	9 (32.1)	21 (33.3)	
CMS daily dose (millions IU)**	5.5 ± 2.4	5.4 ± 2.2	0.793
CMS total dose (millions IU)**	114.4 ± 116.5	113.3 ± 91.5	0.68
CMS duration of treatment, days**	20.3 ± 16.5	21.4 ± 16.6	0.88
Combined treatment, *n* (%)	15 (53.6)	31 (49.2)	0.7
C_ss_ (mg/L)**	2,1 ± 1.4	1.4 ± 1,4	0.011
C_ss_ > 1.25 (mg/L), *n* (%)	18 (64.3)	28 (44.4)	0.093
C_ss_/MIC**	4.2 ± 2.7	3.1 ± 3	0.048
AKI at day 7, *n* (%)	14 (50)	16 (25.4)	0.021
AKI at the EOT, *n* (%)	20 (71.4)	29 (46)	0.025
Length of hospital stay, (days)**	65.2 ± 33.9	71.5 ± 63.2	0.3

^1^ *CKD* chronic kidney disease, ^2^ *ICU* intensive cure unit, ^3^ *CMS* colistin methanesulphonate, ^4^ *IU* international units, ^5^ *C*_*ss*_ colistin plasma concentration at steady-state, ^6^ *MIC* minimal inhibitory concentration, ^7^ *AKI* acute kidney injury, ^8^ *EOT* end of treatment

*median (range)

**mean ± SD

^a^Among urogenital co-morbidities were, renal disease, kidney stones and obstructive uropathy

^b^Among haematological co-morbidities were haemopoietic and lymphoreticular malignances

^c^Among neurological co-morbidities were Alzheimer’s disease, stroke, miastenia gravis, sclerosis and any kind of dementia

Twelve patients (13.2%) presented AKI prior CMS treatment. This factor was not related with 30-day all-cause mortality (*P* = 0,5) or clinical cure (*P* = 1). Impairment of renal function during CMS treatment was observed in 30 (33%) patients on day 7 and in 49 (53.8%) at the EOT. The distribution of AKI on the basis of the RIFLE criteria was 19 (20.9%) R, 9 (9.9%) I and 2 (2.2%) F on day 7 of treatment; and 12 (13.2%) R, 27 (29.7%) I and 10 (11%) F at the EOT. The CMS dose was modified because of AKI in 16 (17.5%) patients. At the end of follow-up, the GFR returned to baseline in 32 (35.1%) patients. Two patients (2.1%) persisted with some grade of impairment of renal function, 15 (16.4%) died and 3 (3.2%) patients had no follow-up information regarding renal function.

## Discussion

Currently, there is a lack of pharmacokinetic and pharmacodynamic data to guide the proper use of colistin in daily clinical practice. We have observed that C_ss_ is not related to clinical cure and 30-day all-cause mortality in a prospective series of patients infected with colistin-susceptible XDR *P. aeruginosa*.

Clinical cure in this series of patients was 79.1% and was similar to those reported in previous studies [9, 10, 23–25]. Factors related with clinical failure were APACHE II score and the presence of nephrotoxicity at the EOT. We highlight the fact that colistin plasma levels were not related with clinical cure. This finding is in some disagreement with recent studies suggesting that higher doses of CMS might be more appropriate for the treatment of infections caused by MDR-GNB [6, 26, 27]. However, some recent clinical experiences did not observe a significant association between the CMS dose and the clinical or microbiological outcomes. Yilmaz et al. studied daily doses of 3 and 6 million IU CMS in the treatment of infections caused by MDR-GNB and observed no differences in the end points [28]. In another study, Zaidi et al. reported that low CMS doses could also be an effective option in the treatment of infections caused by MDR-GNB [29]. The reasons for this apparent lack of relationship between colistin concentrations or doses, and clinical outcome need to be studied in depth in future studies.

In terms of PK analysis, data from the present study are consistent with the results of these previous studies, in the sense that the classic CMS dosage regimens are associated with suboptimal colistin concentrations and suboptimal pharmacokinetic targets for many strains of GN-GNB. However, based on the population model described by Garonzik et al. [6], we defined a set point corresponding to a target AUC_0–24_ of 60 mg.h/L for a *P. aeruginosa* with an MIC value of 0.5 mg/L, and this set point was not related to the clinical outcomes or to 30-day all-cause mortality.

The 30-day all-cause mortality rate in our series was 30.9%. Previous comparative and non-comparative studies have reported similar crude mortality rates [8, 11, 30, 31]. The APACHE II score and the severity of underlying diseases have been previously reported as predictors of mortality in patients treated with CMS for infections caused by MDR-GNB [30]. In patients with ventilator-associated pneumonia due to *P. aeruginosa*, the severity of the clinical presentation has been reported to be the main predictor of mortality [31]. In accordance with these studies, our series showed that the severity of the acute illness and of the underlying diseases were also independent risk factors for death.

An important finding of this study is that although C_ss_ was not found to be a predictor of clinical failure or mortality in the multivariate model, it is interesting that C_ss_ was statistically significant in the univariate model and that higher average C_ss_ values were associated to clinical failure and also with 30-day all-cause mortality. In an attempt to address this finding, we analyzed the ratio of discontinuation or doses adjustment of colistin due to nephrotoxicity and there were no differences between groups. In our opinion this fact have to be studied in larger prospective studies but one possible hypothesis is that higher levels of colistin have been related to higher ratios of nephrotoxicity [20] and maybe nephrotoxicity and not colistin levels is the responsibly of this poor clinical outcome. In fact, the presence of AKI at the EOT was related to 30-day all-cause mortality in a previous study carried by our group [20].

Of note, in our series the presence of nephrotoxicity at the EOT was associated with mortality and clinical failure. Information about the impact of nephrotoxicity during treatment with CMS on clinical outcomes is scarce. The data that have been reported are contradictory: although some studies have reported higher rates of mortality in patients with nephrotoxicity [8, 20], others have not found this association [32, 33]. Our findings suggest that the development of AKI during treatment with CMS seems to be a more important dose-limiting adverse event than previously recognized. According to our previously reported results showing rates of nephrotoxicity of 65–85% with colistin trough concentrations greater than 2.2 μg/mL [20], AKI during treatment with CMS could be controlled by monitoring colistin plasma concentrations.

We acknowledge some limitations of the current study. First is its observational design that makes difficult to draw stronger conclusions. Second, the sample was heterogeneous in terms of the infection source and the administered CMS dose. In this scenario, previous reports have demonstrated that patients with pneumonia responded less favorably to CMS therapy [23, 34] probably due to the poor drug penetration to the epithelial lining fluid. However this is a “real life” series with a significant number of difficult to treat infections that deserve to be studied in the antimicrobial resistance era. Another limitation is that we did not use the loading dose strategy because the protocol study was designed before this practice was recommended in the literature. Although recent published experiences have suggested that this strategy has not been validated with clinical data [32], without this loading dose it may be much longer to reach the steady-state. However colistin levels were extracted at the fourth day of treatment and therefore analyses have been done at the steady state. Fourth is the lack of specific analysis on the MICs of the isolated microorganism in each patient. The efficacy of antimicrobial therapy is not only based on drug exposure or pathogen susceptibility, instead it is an integrated function of both. The lack of this analysis is another possible source of confounding and should be evaluated in future studies. Aware of all these limitations and with the intention of overcoming some of these handicaps, our group has carried out a clinical trial (clinicaltrials.gov number NCT01845246) whose results are pending analysis and publication at this time.

Our findings highlight several issues. First, although it is currently accepted that there is a need for changes in the recommended CMS dosing regimens to achieve higher colistin plasma levels [5, 6], in our study, the C_ss_ was not related to a better clinical outcome. Second, our results suggest that the severity of the infection, the presence of comorbidities and the presence of AKI at the EOT are the most important factors related to the clinical outcome in XDR *P. aeruginosa* infections treated with intravenous CMS. Third, because of these findings, more prospective and homogeneous clinical studies should be conducted to evaluate the benefit of monitoring colistin concentrations in plasma in clinical practice. These studies have to assess the issue of the optimal dose of CMS in order to maximize clinical benefits and minimize toxicities.

## Conclusions

In conclusion in this series of patients, colistin plasma levels were not observed to be related to clinical cure or mortality in patients with XDR *P. aeruginosa* infections treated with CMS. However these finding needs to be confirmed in future studies. Of note, the presence of AKI at the EOT is a risk factor for mortality and clinical failure.

## Abbreviations

AKI: Acute kidney injury
AUC: Area under the curve
CDC: Centers for Disease Control and Prevention
CI: Confidence interval
CKD: Chronic kidney disease
C_max_: Maximum plasma concentrations
C_min_: Minimum plasma concentrations
CMS: Colistimethate sodium
COPD: Chronic obstructive pulmonary disease
C_ss_: Colistin plasma levels at steady-state
EOT: End of treatment
GFR: Glomerular filtration rate
GNB: Gram negative bacteria
HPLC: High performance liquid chromatography
IQR: Interquartile range
IU: International units
MDR: Multidrug-resistant
MDRD: Modification of Diet in Renal Disease equation
MIC: Minimal inhibitory concentration
OR: Odds ratio
ROC curve: Receiving operating characteristic curve
SD: Standard deviation
XDR: Extremely drug-resistant
